# *Pseudomonas fluorescens* Complex and Its Intrinsic, Adaptive, and Acquired Antimicrobial Resistance Mechanisms in Pristine and Human-Impacted Sites

**DOI:** 10.3390/antibiotics11080985

**Published:** 2022-07-22

**Authors:** Myllena Pereira Silverio, Gabriela Bergiante Kraychete, Alexandre Soares Rosado, Raquel Regina Bonelli

**Affiliations:** 1 Laboratório de Ecologia Molecular Microbiana, Instituto de Microbiologia Paulo de Góes, Universidade Federal do Rio de Janeiro (UFRJ), Rio de Janeiro 21941-902, Brazil; myllenapsss@gmail.com (M.P.S.); alexandre.rosado@kaust.edu.sa (A.S.R.); 2 Laboratório de Investigação em Microbiologia Médica, Instituto de Microbiologia Paulo de Góes, Universidade Federal do Rio de Janeiro (UFRJ), Rio de Janeiro 21941-902, Brazil; gabrielabergiante@micro.ufrj.br; 3 Biological and Environmental Sciences and Engineering Division (BESE), King Abdullah University of Science and Technology (KAUST), Thuwal 23955-6900, Saudi Arabia

**Keywords:** *Pseudomonas fluorescens*, antimicrobial resistance, intrinsic resistance, adaptive resistance, acquired resistance

## Abstract

*Pseudomonas* spp. are ubiquitous microorganisms that exhibit intrinsic and acquired resistance to many antimicrobial agents. *Pseudomonas aeruginosa* is the most studied species of this genus due to its clinical importance. In contrast, the *Pseudomonas fluorescens* complex consists of environmental and, in some cases, pathogenic opportunistic microorganisms. The records of antimicrobial-resistant *P. fluorescens* are quite scattered, which hinders the recognition of patterns. This review compiles published data on antimicrobial resistance in species belonging to the *P. fluorescens* complex, which were identified through phylogenomic analyses. Additionally, we explored the occurrence of clinically relevant antimicrobial resistance genes in the genomes of the respective species available in the NCBI database. Isolates were organized into two categories: strains isolated from pristine sites and strains isolated from human-impacted or metal-polluted sites. Our review revealed that many reported resistant phenotypes in this complex might be related to intrinsic features, whereas some of them might be ascribed to adaptive mechanisms such as colistin resistance. Moreover, a few studies reported antimicrobial resistance genes (ARGs), mainly β-lactamases. In-silico analysis corroborated the low occurrence of transferable resistance mechanisms in this *Pseudomonas* complex. Both phenotypic and genotypic assays are necessary to gain insights into the evolutionary aspects of antimicrobial resistance in the *P. fluorescens* complex and the possible role of these ubiquitous species as reservoirs of clinically important and transmissible ARGs.

## 1. Introduction

The genus *Pseudomonas* comprises a wide range of ubiquitous metabolically versatile microorganisms found in diverse ecosystems, including water, soil, and the rhizosphere [1,2]. From a clinical perspective, *Pseudomonas aeruginosa* is the most important and extensively characterized species belonging to this genus [3,4]. Nevertheless, other members of the genus *Pseudomonas* might act as opportunistic pathogens, causing infections mainly in immunocompromised patients or individuals subjected to invasive medical procedures. For instance, *Pseudomonas fluorescens* and related species have been reported to cause bloodstream, urinary, pulmonary, cerebrospinal, joint-fluid, skin, and soft-tissue infections [5,6,7,8,9,10,11,12,13,14]. Furthermore, epidemiological studies of nosocomial infections have revealed that resistant *P. fluorescens* strains can be transported into hospitals by water or other materials used in medical procedures, as well as by insects such as moth flies [15,16,17,18,19].

The description of new species and the reclassification of those previously defined as *Pseudomonas*, such as *Burkholderia*, *Ralstonia*, *Comamonas*, *Acidovorax,* and *Hydrogenophaga*, is a continuous process [20,21]. Based on 16S rDNA analysis and multi-locus sequence analysis (MLSA), the genus comprises three main lineages recognized as *P. aeruginosa*, *P. fluorescens,* and *P. pertucinogena*, which have been divided into fourteen groups [22]. The *P. fluorescens* complex comprises eight groups occupying various ecological niches, namely *P. fluorescens*, *P. gessardii*, *P. fragi*, *P. mandelii*, *P. koreensis*, *P. jessenii*, *P. corrugata*, and *P. chlororaphis* [22,23].

Over the last 30 years, antimicrobial resistance has been described in isolates of the *P. fluorescens* complex obtained from non-medical sources. However, the phenomenon of intrinsic resistance in *Pseudomonas* poses a challenge when studying the role of these species as environmental reservoirs of transferable resistance genes. This review compiles literature reports on antimicrobial resistance detected in strains belonging to the *P. fluorescens* complex occupying different environmental niches, thus establishing a theoretical basis for the evaluation of the possible role of these bacteria as environmental reservoirs of antimicrobial resistance genes.

## 2. Material and Methods

### 2.1. Whole-Genome Sequencing Analysis

A phylogenomic approach based on genomes from type strains of all species already described for the genus *Pseudomonas* was used to identify species belonging to the *P. fluorescens* complex. *Pseudomonas* spp. with valid publications and correct names (excluding synonyms) according to the List of Prokaryotic names with Standing in Nomenclature (https://lpsn.dsmz.de/genus/pseudomonas, accessed on 26 October 2021) were included in this analysis.

Out of the 269 retrieved species, 243 were selected for further analysis based on the availability of a published type strain genome. After generating a phylogenomic tree using the Type Strain Genome Server (https://tygs.dsmz.de/ accessed on 26 October 2021), 98 species associated with the *P. fluorescens* complex (Figure S1) were identified. To understand the composition of the groups, digital DDH estimation (dDDH) was calculated with the GGDC 3.0 web service (http://ggdc.dsmz.de accessed on 26 October 2021) using the BLAST+ alignment tool and formula 2 (identities/HSP length). The dDDH data were plotted on a heatmap (Supplementary Figure S1) and detailed in Table S1. Species with more than 31.8% dDDH were considered to belong to the same group [23].

Data obtained from whole-genome sequencing (WGS) of species belonging to the *P. fluorescens* complex were further analyzed. WGS data from these species were obtained from the PATRIC database (https://www.patricbrc.org accessed on 28 November 2021), after which the available data were filtered according to genome status (WGS; Complete), assembly accession (empty spaces were removed), and isolation source (empty spaces were also removed), resulting in 619 genomes. The genomes were downloaded using the NCBI assembly accession with the ncbi-genome-download software (version 0.3.1; https://github.com/kblin/ncbi-genome-download accessed on 28 November 2021). Next, acquired antimicrobial resistance genes (ARGs) were identified using ABRicate version 1.0.0 (https://github.com/tseemann/abricate accessed on 28 November 2021) and the ResFinder database (https://cge.cbs.dtu.dk/services/ResFinder/ accessed on 28 November 2021) (Table 1).

### 2.2. PubMed and Google Scholar Research Approach

A comprehensive review was conducted using the names of species belonging to the *P. fluorescens* complex as keywords. These included the species identified via the phylogenomic analysis presented in this study (Figure S1), as well as a recent phylogenetic MLSA based on four genes [22,23,24], alongside the words “antibiotics”, “antimicrobial”, “susceptibility”, or “resistance” in the PubMed platform. Google Scholar was used when a given study was unavailable in the PubMed database. No time frame was defined. Overall, the selected studies were categorized based on the origin of the isolates and were critically evaluated in light of current knowledge on intrinsic, adaptive, and acquired resistance. The recovered data was organized in two tables. Table 1 summarizes the available literature describing antimicrobial resistance phenotypes of isolates collected in pristine environments. In contrast, Table 2 focuses on isolates from urban, human-impacted, or metal-polluted sites. Both tables indicate the resistance phenotypes typically recognized as intrinsic in non-fermentative gram-negative bacteria or *P. aeruginosa*. Genotypic characteristics were also included when available.

## 3. Antimicrobial Resistance in the *P. fluorescens* Complex

### 3.1. Intrinsic Resistance

The European Committee on Antimicrobial Susceptibility Testing (EUCAST) guideline “Expected Resistant Phenotypes” (http://www.eucast.org accessed on 12 May 2022) reports that non-fermentative gram-negative bacteria are intrinsically resistant to benzylpenicillin, first- and second-generation cephalosporins, glycopeptides, lipoglycopeptides, fusidic acid, macrolides, lincosamides, streptogramins, rifampicin, and oxazolidinones. Concerning other antimicrobials not cited above, the same document stated that *P. aeruginosa* is expected to be resistant to ampicillin and amoxicillin, as well as their combinations with β-lactamase inhibitors, ceftriaxone, cefotaxime, ertapenem, chloramphenicol, selected aminoglycosides such as kanamycin and neomycin, trimethoprim, tetracycline, and tigecycline [25]. Knowledge of the intrinsic resistance of *P. fluorescens* is still limited; however, the trends appear to be similar to those of *P. aeruginosa*.

Regarding β-lactams, Rocha et al. evaluated 39 endophytic strains of *Pseudomonas* sp. isolated from a metal-accumulating plant, after which they correlated the resistance phenotypes with the distribution of strains in a dendrogram [26]. The authors recognized three clusters. The first cluster, composed of a few isolates from five different species, was associated with resistance to ampicillin and amoxicillin, and was susceptible to most of the β-lactams tested. The second cluster, formed mainly by *P. koreensis* (*P. koreensis* group, n = 10) and *Pseudomonas simiae* (*P. fluorescens* group, n = 4), showed resistance to ampicillin, amoxicillin, amoxicillin–clavulanic acid, and cefotaxime. Many isolates were also resistant to aztreonam, sulfamethoxazole–trimethoprim, and chloramphenicol. In contrast, the third cluster formed mainly by *Pseudomonas sabulinigri* (n = 11) included isolates that were resistant to the same β-lactams as in the second cluster, in addition to piperacillin and piperacillin–tazobactam. Some isolates belonging to the third cluster also displayed resistance to cefepime and ceftazidime but were susceptible to other classes of antimicrobial agents. Acquired resistance genes for β-lactams (*bla*_SHV_, *bla*_TEM_, *bla*_CTX-M_, *bla*_GES_, *bla*_KPC_, *bla*_VIM_, *bla*_IMP_, *bla*_OXA-2-like_, *bla*_OXA-10-like_, *bla*_OXA-30-like_), sulfonamides (*sul1*), and chloramphenicol (*cat*), as well as the integrase genes *intI1* and *intI2*, were not detected in these strains, supporting the hypothesis that the phenotypes were associated with intrinsic features [26].

The production of an inducible chromosomal β-lactamase called AmpC contributes to *Pseudomonas* resistance to most penicillins and to first- and second-generation cephalosporins (such as cefoxitin and cefuroxime). In *P. aeruginosa*, de-repression of this gene can result in resistance to antipseudomonal penicillins, oxyiminocephalosporins, and cefepime [27,28]. Accordingly, among several β-lactamase genes searched, only the chromosomal class C β-lactamase gene *bla_AmpC_* was detected in two *P. koreensis* isolates from urban wastewater treatment plants in Italy, showing low susceptibility to several β-lactams [29].

Recently, acquired β-lactamase genes have not been identified among isolates belonging to the *P. fluorescens* complex resistant to aztreonam and carbapenems isolated from chicken meat in Norway. Besides genes encoding efflux pumps, the isolates carried *bla_AmpC_* and the penicillin-binding protein gene *mrcA*; however, mutations in these genes or their promoters have not been addressed. In some isolates, the authors also reported the detection of the *pbpC* gene, which encodes a PBP3 homolog [30]. PBP3, a penicillin-binding protein encoded by *ftsI*, is the target of aztreonam, and mutations in this gene may affect the activity of the drug [31]. Further studies are necessary to investigate the relevance of this gene in aztreonam resistance.

The intrinsic resistance of *P. aeruginosa* to chloramphenicol, trimethoprim, and tetracyclines can be ascribed to the presence of chromosomally expressed resistance-nodulation-division (RND)-type multidrug efflux systems on the cell surface. Notably, multidrug-resistance (MDR) efflux pumps are conserved in different microorganisms, which are likely involved in the extrusion of many toxic compounds [32]. For example, environmental *P. aeruginosa* strains isolated prior to the discovery of quinolones can extrude this class of antimicrobial agents, suggesting that antimicrobial extrusion is not the primary function of some efflux pumps [33,34]. Likewise, the RND-type efflux pump MexAB–OprM has been detected in a β-lactam resistant *Pseudomonas* strain submitted to WGS [30].

In this context, genes encoding an RND efflux pump for polycyclic aromatic hydrocarbons (PAHs) termed EmhABC have been described in the *P. fluorescens* strain cLP6a [35]. Disruption of the *emhB* gene increased the activity of chloramphenicol and nalidixic acid, but not tetracycline, erythromycin, trimethoprim, or streptomycin, suggesting a more limited spectrum of substrates compared to other RND pumps [35,36]. Later, it was suggested that an alternative EmhABC efflux pump conferred resistance to ampicillin, chloramphenicol, tetracycline, ethidium bromide, and crystal violet in the *P. fluorescens* strain 2P24, which was isolated from wheat roots [37]. Complementary studies showed that incubation temperature and other physicochemical factors may affect EmhABC activity in *P. fluorescens* cLP6a [38,39].

Likewise, the knockout of some putative transporters increased the susceptibility of *Pseudomonas protegens* (*P. protegens* group) to rifampicin, among other toxic compounds [40]. Also, genes encoding many efflux-pump proteins, β-lactamases, and a macrolide glycosyltransferase have been described in the genome of the plant growth-promoting bacterium *Pseudomonas* sp. UW4 (*P. jessenii*, according to the authors; *P. jessenii* group). This strain was resistant to ampicillin, erythromycin, and novobiocin [41].

Aminoglycosides are cationic drugs, and the incubation temperature might affect their activity. Papapetropoulou et al. reported that temperatures higher than 37 °C lowered the minimum inhibitory concentration (MIC) of *P. fluorescens* to gentamicin and amikacin, suggesting that higher temperatures promote changes in cell-wall lipids, which increases the permeability to these aminoglycosides [42]. *P. aeruginosa* harbors the chromosomally encoded aminoglycoside phosphotransferase APH(3′)-IIb, having kanamycin and neomycin as substrates [25,42,43]. However, in a search for “APH(3′)” in the NCBI database, only a few results report this gene associated with other *Pseudomonas* species, including *P. fluorescens*.

Colistin is a cationic drug that is used to treat *P. aeruginosa* infections [25,44]. Still, all five *P. koreensis* isolates obtained from urban wastewater-treatment plants in Italy were resistant to this polymyxin [29]. Moreover, many *Pseudomonas* species with plant-beneficial properties, such as *P. protegens* and *P. chlororaphis*, have been reported as intrinsically resistant to cationic compounds [45]. According to the authors, the phenotype is dependent on the presence of O-specific side chains on the cell surface [45]. Among clinical isolates of *P. aeruginosa*, adaptive resistance to polymyxins can occur due to the addition of 4-amino-4-deoxy-L-arabinose (Ara4N) to the lipid A moiety of lipopolysaccharide through induction of the *arn* operon under the control of two-component regulatory systems [46]. Recently, six genes related to colistin resistance (*emrA*, *lpxA*, *lpxD*, *pgsA*, *phoP*, *phoQ*), but not the plasmid-mediated *mcr*, have been detected in the genome of colistin-resistant *Pseudomonas* spp. obtained in the Norwegian food chain [30].

### 3.2. Antimicrobial Resistance in Pristine Sites

Atypical antimicrobial resistance profiles in *Pseudomonas* sp. obtained from pristine sites have been reported in many studies. Shivaji et al. (1989) obtained 10 isolates of *Pseudomonas* spp. (including *P. fluorescens*) from Antarctic soil samples, which were susceptible to kanamycin, gentamicin, tobramycin, polymyxin B, tetracycline, rifamycin, colistin, streptomycin, and nalidixic acid [47]. At that time, considering that these strains grow at low temperatures (4 °C), authors suggested that such a distinct phenotype compared to mesophilic *Pseudomonas* strains may have resulted from adapting to harsh conditions [47]. Later, the psychrophilic species *P. antarctica*, *P. meridiana*, and *P. proteolytica* (*P. gessardii* group) obtained from cyanobacterial mats in Antarctica were characterized [48]. Curiously, *P. antarctica* was also susceptible to antimicrobial agents considered ineffective against *P. aeruginosa* due to intrinsic resistance, such as penicillin, ampicillin, chloramphenicol, sulfamethoxazole–trimethoprim, erythromycin, kanamycin, and tetracycline. In contrast, *P. meridiana* and *P. proteolytica* were resistant to all the antimicrobials mentioned above, except for kanamycin and tetracycline. Similar to *P. aeruginosa*, the three *Pseudomonas* strains tested were resistant to trimethoprim [25,48]. More recently, Orellana-Saez et al. isolated the *Pseudomonas* sp. strain MPC6 (closely related to *P. fluorescens*, according to the authors) of a soil sample from Deception Island (Antarctica) [49]. This isolate was susceptible to antimicrobials that are ineffective against most of the *Pseudomonas* analyzed and had a similar resistance profile to the environmental isolates *P. putida* KT2440 and *P. antarctica* (*P. fluorescens* subgroup). Genes encoding antibiotic-inactivation enzymes found in the genome of reference strains *P. aeruginosa* PA7 and *P. aeruginosa* PAO1 such as aminoglycoside phosphotransferases (APHs), chloramphenicol acetyltransferases (CATs), bleomycin-binding proteins, and β-lactamases, were absent in the genome of *Pseudomonas* sp. MPC6. The genome of *Pseudomonas* sp. MPC6 also lacked genes encoding modifications in cell-wall charges that are reported as determinants of antimicrobial resistance. However, the genome was well equipped with efflux pumps [49]. An uncommon susceptible phenotype to penicillin, kanamycin, neomycin, and tetracycline was also detected in *Pseudomonas* sp. strain AHD-1 (closely related to *P. azotoformans*, *P. gessardii*, and *P. libanensis*, according to the authors), which was isolated from wastewater in Tunisia [50].

Furthermore, recent studies have reported *P. fluorescens* members that are resistant to clinically relevant antibiotics such as piperacillin, aztreonam, ceftazidime, carbapenems, and colistin. These strains were isolated from diverse environments such as Antarctica soil samples, rhizosphere of desert plants in Atacama, and calcite moonmilk deposits from caves in the Czech Republic [51,52,53]. Known acquired resistance mechanisms associated with these unexpected phenotypes have not been detected, although genomic islands and other likely acquired mobile genetic elements have been reported in *P. fildesensis* (*P. fluorescens* group) [51]. Therefore, further studies are needed to characterize the pathogenic potential and the presence of transmissible ARGs in such *Pseudomonas* sp. strains.

Although Antarctica is the most remote continent in the world, antimicrobial resistance may be transferred to this region due to the migration of animals and humans. Recently, Na et al. evaluated isolates from animal feces, soil, and sediments with varying human and animal impacts in the Fildes Peninsula, Antarctica [54]. *Pseudomonas* was the dominant genus that showed resistance to sulfamethazine, and a strong correlation between mobile genetic elements and antimicrobial resistance genes was recognized, considering isolates of different genera included in the study [54]. In contrast, in a study that included isolates either from human-impacted or pristine sites in Antarctica, most of the strains displaying multi-resistance were collected from areas without human intervention, suggesting that antimicrobial resistance is likely a natural and ancestral process [55]. Yet, in the characterization of two multidrug-resistant isolates belonging to the *P. fluorescens*, Marcoleta et al. (2022) reported that although these strains lack genes found in the reference *P. aeruginosa* PA7 strain, they showed a higher number of genes associated with ATP-binding cassete (ABC) and small multidrug resistance (SMR) efflux pumps [55]. Genes for putative β-lactamases have also been detected, including a homolog of LRA-3 β-lactamase, which was previously described in soil metagenomic DNA from Alaskan soil [55,56].

### 3.3. Antimicrobial Resistance in Human-Impacted Sites

In human-impacted sites, the selective pressure caused by antimicrobial pollution may promote the dissemination and persistence of acquired resistance mechanisms. Chow et al. exposed one strain of *P. aeruginosa* and one of *P. protegens* to kanamycin, tetracycline, or ciprofloxacin at 1/10 of the MIC in a serial streaking over 40 passages, thus mimicking environmental pollution with antimicrobial agents. Higher MICs and increased genome changes were detected in *P. protegens*, suggesting that this type of antimicrobial pollution might generate new resistant strains [57].

Compared to contemporary samples, ancient and well-conserved samples are powerful tools to measure the degree to which the rates of antimicrobial resistance have changed over the years. Addressing this issue, Lugli et al. characterized a *P. veronii* (*P. fluorescens* group) strain isolated from a frozen and mummified human body found in an Italian Alpine glacier [58]. Screening for ARGs revealed an abundance of putative β-lactamases, glycopeptide-resistance proteins, ABC transporters, and major facilitator superfamily (MFS) efflux pump. Notwithstanding, modern strains of *P. veronii* harbor 24% more ARGs than the ancient strain *P. veronii*, which might be due to horizontal gene transfer (HGT), accounting for the rapid spread and persistence of antimicrobial resistance determinants in the environment [58].

As an example of exceptional resistance phenotypes identified in highly human-impacted sites, *P. fluorescens* strains resistant to clinically available antimicrobial agents, such as piperacillin-tazobactam, ceftazidime, cefepime, imipenem, meropenem, gentamicin, and ciprofloxacin, were isolated from the multinational Danube River [59,60].

#### 3.3.1. Metal-Polluted Sites

Metal resistance can be accompanied by antimicrobial resistance, as suggested by several references listed in Table 3 [26,61,62,63,64,65,66]. Metals are not easily degraded and occur in various environments, especially those receiving hospital and industrial effluents, as well as mining areas [67]. Furthermore, metal pollution causes a persistent selective pressure that favors the development and transmission of antimicrobial resistance traits [68]. There are two known mechanisms through which metal and antimicrobial resistance are co-selected. So-called “co-resistance” refers to the presence of metal- and antimicrobial-resistance determinants encoded in the same mobile genetic element, whereas “cross-resistance” refers to the same mechanism conferring resistance to both metals and antimicrobial agents (e.g., efflux pumps) [68,69]. In addition to the data on *P. fluorescens* found in this review, previous studies have evaluated metal and antimicrobial resistance in hospital and environmental isolates of members of the genus *Pseudomonas* [67,70,71,72,73].

Ramos et al. detected *P. saponiphila* (n = 13; *P. chlororaphis* group), *P. humanensis* (n = 5) and *P. asiatica* (n = 2) (*P. putida* group), and *P. aeruginosa* (n = 3) in water samples obtained from the state of São Paulo and Brasília (Brazil) [73]. Most of these isolates were resistant to heavy metals and clinically relevant antimicrobial agents, such as the β-lactams piperacillin-tazobactam, ceftazidime, cefepime, imipenem, meropenem, and aztreonam, as well as the quinolones ciprofloxacin, levofloxacin, and norfloxacin, and the aminoglycosides gentamicin and tobramycin. The ARGs *bla*_GES_ (β-lactam resistance), *tetB* (tetracycline resistance), *qnrS* and *qepA* (quinolone resistance), and *aac(3′)-IIa* and *ant(2″)-Ia* (aminoglycoside resistance) were identified for the first time in *P. saponiphila*. Even so, plasmids were not detected, suggesting that the identified genes could be located in the chromosome. These findings suggested that the resistant phenotype for most of the antimicrobial agents and heavy metals analyzed might be attributed to alternative mechanisms that were not evaluated, such as efflux pumps of the RND superfamily [73].

#### 3.3.2. Other Reservoirs of Human Importance

Resistant *P. fluorescens* strains have been found in food products such as chicken and camel meat, salad vegetables, fish and mushroom farms, as well as in cheese [30,74,75,76,77,78,79,80,81]. Notably, *Pseudomonas* spp. is one of the main microorganisms causing food spoilage [30,82,83,84,85]. Likewise, Poirel et al. isolated *P. synxantha* (*P. fluorescens* group) from chicken meat, which harbored a likely-acquired chromosomal metallo-β-lactamase PFM-1 [80]. PFM-1 showed high amino-acid identity with Sfh-1 and CphA-1 carbapenemases, which were initially reported in species of *Serratia* and *Aeromonas*, respectively. Variants of PFM-1 were also detected in *P. libanensis* (PFM-2) and *P. fluorescens* (PFM-3), suggesting that the *P. fluorescens* group may behave as reservoir of PFM-like encoding genes [80]. Recently, one isolate of *P. fluorescens* harboring the β-lactamase *bla*_SHV_ was identified in Benin. The isolate was resistant to amoxicillin, amoxicillin-clavulanic acid, ceftriaxone, cefotaxime, ertapenem, imipenem, aztreonam, gentamicin, and ciprofloxacin [86].

Members of the *P. fluorescens* complex have also been recognized as veterinary pathogens, causing infections in bovines, canines, dolphins, fish, wild animals, and even frog oocytes. Common resistance phenotypes observed in these strains include tetracycline, chloramphenicol, sulfamethoxazole-trimethoprim, amoxicillin, amoxicillin–clavulanic acid, cefoxitin, cefotaxime, and ticarcillin resistance. Moreover, most of these animals were living in environments under the human influence [87,88,89,90,91,92,93].

Due to the clinical importance of *P. aeruginosa*, breakpoints for effective antimicrobial agents against this pathogen are available in the Clinical and Laboratory Standards Institute (CLSI) and EUCAST guidelines. The β-lactams ticarcillin, piperacillin, ceftazidime, cefepime, cefiderecol, ceftolozane, aztreonam, imipenem, doripenem, and meropenem, some of which are associated with β-lactamase inhibitors, as well as the quinolones ciprofloxacin and levofloxacin, the aminoglycosides gentamicin, amikacin, tobramycin, and polymyxins colistin or polymyxin B, should be effective against this microorganism [94,95]. Table 1 and Table 2 list the *P. fluorescens* isolates with resistant phenotypes to one or more of the drugs listed above. However, few reports have described their genetic features, and further studies identifying ARGs in *P. fluorescens* are needed to infer whether the resistance profile was acquired or adaptive. Still, β-lactamases are the most reported among the ARGs listed in Table 2 and Table 3.

### 3.4. Horizontal Gene Transfer (HGT) of Antimicrobial Resistance

Possible HGT from hospitals and health facilities to the environment is a serious public health concern. Forsberg et al. analyzed the transfer of resistance determinants between soil and clinical bacteria. Resistance genes present in the environmental bacterium *Pseudomonas* sp. K94.23 (according to the authors, a member of the *P. fluorescens* complex) shared a complete nucleotide identity with clinical pathogens [96]. Likewise, Herrick et al. suggested that transmissible plasmids from environmental *Pseudomonas* that confer resistance to tetracycline (commonly used in agriculture) would cause the persistence of co-carried genes that confer resistance to clinically available antimicrobial agents such as gentamicin, ticarcillin, and ciprofloxacin [97]. In the 1990s, Chandrasekaran et al. suggested that even viable but non-culturable *P. fluorescens* can transfer plasmids to other bacteria in marine environments [98]. The same group also reported the transference of an MDR plasmid (pSCL) from rifampicin-resistant *P. fluorescens* isolated from polluted soil to *E. coli* and *P. putida*. It appears that pSCL conferred rifampicin resistance to the transformants, presumably through an efflux pump [99].

Efficient transference of plasmids carrying resistance genes to chloramphenicol (*cat*) [100] and colistin (*mcr*-variants) have been reported for *P. putida* and/or *P. aeruginosa* [101,102,103]. Regarding *P. fluorescens*, the gene *mcr-1* was detected in one isolate from a community/household environment in the Republic of Congo [104]. A gene encoding BIC-1, a class A carbapenemase capable of hydrolyzing penicillins, cephalosporins (except ceftazidime), and carbapenems, was detected in the chromosome of a *P. fluorescens* isolate from the Seine River (France). Three months later, the β-lactamase gene was also found in the chromosomes of two other *P. fluorescens* isolates from the same site [105]. Furthermore, the class B metallo-β-lactamase IMP-22, encoded by the *bla*_IMP-22_ gene, located in a class 1 integron and capable of hydrolyzing narrow and extended-spectrum β-lactams, was detected in two strains of *P. fluorescens* from urban wastewater, as well as in a clinical isolate of *P. aeruginosa* in Italy [64].

The risk of human infection by exposure to contaminated rivers was illustrated by a case of a patient admitted to an intensive care unit for near-drowning in a river in France, who was colonized and infected by carbapenem-resistant bacteria of probable environmental origin. Among the isolates characterized, six strains belonging to the *P. fluorescens* complex collected from the river had the same carbapenem-resistant phenotype as a *P. fluorescens* strain colonizing the patient’s respiratory tract [106]. In another country, an isolate of *P. cedrina* (*P. fluorescens* group) was identified in water bodies in Los Angeles, which was resistant to cefotaxime, meropenem, and imipenem [107]. However, none of these studies reported carbapenemase production, suggesting that other genotype features, transmissible or not, might be responsible for the observed phenotype.

In 2012, Maravić et al. published the first report of a TEM-type ESBL in *P. fluorescens* [108]. The *bla_TEM_*_-116_ gene was present in the chromosome of isolates from a Croatian bay highly impacted by agricultural, industrial, and municipal effluents. In the same study, out of 185 *P. fluorescens* isolates investigated, 70 presented a multidrug resistant phenotype, with the highest resistance rates for cefotaxime, ceftazidime, meropenem, aztreonam, and tetracycline [108]. Later studies have reported the presence of *bla_TEM_* in isolates belonging to the *P. fluorescens* group in South Africa and India [109,110].

Some results from studies whose focus was not on the genus *Pseudomonas* are worth mentioning. *Pseudomonas* was one of the most abundant genera recovered under selective pressure with cefotaxime or imipenem from Lake Bolonha in the Brazilian Amazon. Among 37 *Pseudomonas* strains displaying the above-mentioned resistance phenotypes (species were not determined), 25 carried the likely acquired β-lactamase genes *bla*_TEM_, *bla*_SHV_, *bla*_CTX_, *bla*_IMP_, and *bla*_VIM_ either alone or in combination. Many of these isolates were also resistant to non-β-lactams such as gentamicin [111]. Similarly, Chakraborty et al. reported that *Pseudomonas* was the most abundant genus contributing to the occurrence of ARGs, including multidrug-efflux pumps, glycopeptide, bacitracin, tetracycline, and aminoglycoside-resistance genes, in the Lonar soda lake (India) [112].

### 3.5. Research of Resistance Genes in Genome Public Databases

Congruent with the data collected in the scientific literature, the analysis conducted herein identified a small number of genomes of species belonging to the *P. fluorescens* complex (n = 17, 2.7%) carrying one or more transferable ARG. Genes associated with resistance to β-lactams, aminoglycosides, phenicol, fosfomycin, sulfamethoxazole, or tetracycline classes have been found in isolates of different species, which were recovered from variable sources and countries (Table 3).

**Table 1:** Isolates of the *Pseudomonas fluorescens* complex obtained from pristine environments and their resistance phenotypes/genotypes.

Isolation Site	Isolates Identified and Characterized as Belonging to the *Pseudomonas fluorescens* Complex (Group)	Phenotype/Genotype (Number of Isolates)	Ref.
Soil/Schirmacher Oasis, Antarctica	*P. fluorescens* (*P. fluorescens*)	Resistance to **P**, **AM**, CB, **E**, **VAN**, **TMP**, and **BAC**, and to the antifungal **NY**. Susceptibility to **C** varied (undefined number of isolates) ^A^	[47]
Cyanobacterial mat/McMurdo region, Antarctica	Description of *P. antarctica* strain CMS35^T^ (*P. fluorescens*), *P. meridiana* strain CMS38^T^ (*P. gessardii*), and *P. proteolytica* CMS64^T^ (*P. gessardii*)	Resistance to **TMP** and **FUZ** in *P. antarctica* (1); resistance to **P**, **AM**, **AMX**, CB, **E**, **LIN**, **C**, **TMP**, **SXT**, GM, CL, PB, **BAC**, FM, **FUZ**, and **NY** in *P. meridiana* (1) and resistance to **P**, **AM**, **AMX**, CB, **E**, **LIN**, **C**, **TMP**, **SXT**, **FUZ**, **NY**, **BAC**, FM, and NFZ in *P. proteolytica* (1) ^A^	[48]
Rhizosphere of Amaranth/Northwestern Indian Himalayas	*Pseudomonas* sp. NARs9 (closely related to *P. lurida*, according to the authors)	Resistance to **AM**, **P**, PB, and **C** (1) ^B^	[113]
Drinking water from karstic ecosystems/Le Havre, France	*P. fluorescens* (*P. fluorescens*) and *P. brenneri* (*P. gessardii*)	*P. fluorescens* (6) and *P. brenneri* (1) were resistant to **CF**, **AMX**, **AMC**, **CTX**, CFS, TIC, TIM, **C**, and **SXT**. Resistance to ATM, FOS, and NA was frequent among the isolates ^A^	[114]
Soil/Isla de los Estados, Ushuaia, Argentina	Description of *P. yamanorum* strain 8H1^T^ (*P. gessardii*)	Resistance to **P**, **OX**, **CF**, **CXM,** **SAM**, **CTX**, CAZ, **E**, **CM,** **TEC**, **VA**, **C**, **SXT**, and CL (1) ^A^	[115]
Brook sediment/Whalers Bay, Deception Island, Antarctica Ornithogenic soil/Galindez Island, Antarctica	Metal-resistant *P. migulae* (*P. mandelii*)*, P. gessardii* (*P. gessardii*), and *P. fluorescens* (*P. fluorescens*)	*P. migulae* was intermediate to CIP and TM, resistant to GM, NB, **LIN**, **AM**, **TE**, **C**, **VA**, **E**, **CZ** (1) ^A^ *P. gessardii* was intermediate to GM and resistant to NB, **LIN**, **TE**, **AM**, **C**, **VA**, **E**, and **CZ** (1) ^A^ *P. fluorescens* was intermediate to **CZ** and **E**, resistant to NB, **LIN**, **AM**, and **VA** (1) ^A^	[116]
Soil from the northern deglaciated part of Ulu Peninsula/James Ross Island, Antarctica	Description of *P. gregormendelii* strain CCM 8506^T^ (closely related to *P. migulae*, according to the authors)	Resistance to β-lactams TIC, TIM, CAZ, and ATM (2) ^A^	[117]
Rhizospheres of wild cranberry plants/Cape Cod National Seashore, Massachusetts, USA	Draft genome of *Pseudomonas* sp. strain MWU12-2534b (closely related to *P. protegens*, according to the authors)	Detection of efflux-pump genes, β-lactamases, aminoglycoside N(6′)-acetyltransferase, and fluoroquinolone resistance (1)	[118]
Soil sample/King George Island, Antarctica	Description of *fildesensis* KG01^T^ (*P. fluorescens*)	Resistance to CAZ and IPM ^C^	[51]
Rhizosphere of desert bloom plant/Atacama, Chile	Description of *P. atacamensis* M7D1^T^ (*P. koreensis*)	Intermediate to MEM; resistance to **CTX**, CAZ, **AM**, and **SAM** ^A^	[52]
Calcite moonmilk deposits from caves/Moravian Karst, Czech Republic	Description of *P. karstica* HJ/4^T^ and *P.* *spelaei* SJ/9/1^T^ (*P. gessardii*)	Strain HJ/4^T^: resistance to ATM, PIP, and TZP. Strain SJ/9/1^T^: resistance to ATM and CL ^D^	[53]
Antartic Peninsula Soil	Pseudomonas ArH3a and Pseudomonas YeP6b (*P. fluorescens*)	Strains resistant against 9 to10 antimicrobial agents ^A^ with arbitrary breakpoints. Detection of genes encoding ABC and SMR efflux pumps	[55]

Antimicrobials to which *P. aeruginosa* is considered intrinsic resistant are indicated in bold. Antimicrobials in both bold type and underlined are generally recognized as ineffective against non-fermentative gram-negative bacteria. ^T^: type strain; ^A^: disc-diffusion method; ^B^: minimum inhibitory concentration (MIC) by agar dilution; ^C^: MIC by Etest; ^D^: Mikrolatest MIC based on the Neferm kit (Erba Lachema, Czech Republic). AM: ampicillin; AMC: amoxicillin–clavulanic acid; AMX: amoxicillin; ATM: aztreonam; BAC: bacitracin; C: chloramphenicol; CAZ: ceftazidime; CB: carbenicillin; CF: cephalothin; CFS: cefsulodin; CZ: cefazolin; IPM: imipenem; MEM: meropenem; CIP: ciprofloxacin; CL: colistin; CM: clindamycin; CTX: cefotaxime; CXM: cefuroxime; E: erythromycin; FM: nitrofurantoin; FOS: fosfomycin; FUZ: furazolidone; GM: gentamicin; LIN: lincomycin; NA: nalidixic acid; NB: novobiocin; NFZ: nitrofurazone; NY: nystatin; OX: oxacillin; P: penicillin; PB: polymyxin B; SAM: ampicillin–sulbactam; SXT: sulfamethoxazol–-trimethoprim (or cotrimoxazole); TE: tetracycline; TEC: teicoplanin; TIC: ticarcillin; TIM: ticarcillin–clavulanic acid; PIP: piperacillin; TZP: piperacillin–tazobactam; TMP: trimethoprim; TM: tobramycin; VA: vancomycin.

**Table 2:** Isolates of the *Pseudomonas fluorescens* complex obtained from human-impacted or metal-polluted environments and their resistance phenotypes/genotypes.

Isolation Site	Isolates Identified and Characterized as Belonging to the *Pseudomonas fluorescens* Complex (Group)	Phenotype/Genotype (Number of Isolates)	Ref.
Sewage from sixteen sites/Casablanca, Morocco	Heavy metal-resistant *P. fluorescens* (*P. fluorescens*)	Notable resistance to all antibiotics tested, including **AM**, **AMX**, **CRO**, **RIF**, **C**, **TE**, CIP, and SPIR. (Undefined number of isolates) ^A^	[61]
Subtropical and temperate soils from maize fields/Sikkim Himalaya, India	*P. corrugata* (*P. corrugata*)	Resistance to **P**, **AM**, and CB at high concentrations (2) ^B^	[119]
Sea water	Arsenic-resistant *P. fluorescens* strain MSP3 (*P. fluorescens*)	Resistance to **AM**, **RIF**, **NB**, and **BAC **(1) ^C^	[120]
Kiwi-fruit plants/Korea and Japan	*P. marginalis* (*P. fluorescens*)	Streptomycin-resistance genes (*strA* and *strB*) (1)	[121]
Soil artificially polluted with 1000 mg chromate (Cr(VI)) kg^−1^	*P. corrugata* (*P. corrugata*)	Resistance to **AM**, **CF**, **CRO**, **C**, **BLE**, and FOS (2) ^D^	[63]
Hydrotherapy swimming pool/Northwestern Greece	*P. fluorescens* (*P. fluorescens*)	Resistance to TIC, TIM, AZT, **SUT**, FM, and TM; intermediate resistance to TZP and IPM (2) ^E^	[122]
Rape roots of *Brassica napus* in heavy metal-contaminated soils/Nanjing, China	*P. fluorescens* (*P. fluorescens*)	Resistance to **AM**, **K**, STS, and SPT (1) ^B^	[62]
Urban wastewater/L’Aquila, Italy	*P. fluorescens* (*P. fluorescens*)	Presence of the gene *bla*_IMP-22_, which encodes the metallo-β-lactamase IMP-22, capable of hydrolyzing narrow- and extended-spectrum β-lactams (2)	[64]
Blowhole, gastric fluid, and feces of *Tursiops truncatus* dolphins from estuarine waters/Charleston and Indian River Lagoon, USA	*P. fluorescens* (*P. fluorescens*)	Frequent resistance to **AM**, **AMC**, **CF**, **TE**, **E**, **C**, **SUT**, and FM. Less-frequent resistance to PIP and EN (82) ^C^	[123]
Soil contaminated with wastewater/Sfax, Tunisia	Description of *Pseudomonas* sp. strain AHD-1 (closely related to *P. azotoformans*, *P. gessardii*, and *P. libanensis*, according to the authors)	Resistance to **E** and **C** (1) ^C^	[50]
Water from the Seine River/Paris, France	*P. fluorescens* (*P. fluorescens*)	Production of BIC-1, an Ambler class A carbapenemase capable of hydrolyzing penicillins, carbapenems, and cephalosporins (except CAZ). Resistance to TIC, TIM, PIP, and ATM, among others (1) ^E^	[105]
Seawater/Algiers, Algeria	*P. fluorescens* (*P. fluorescens*)	All 7 isolates tested were resistant to **AMX**, **AMC**, and **FOX**; 6 to **CTX**, TIC, TIM, and NA; 5 to CFS; 4 to **TMP**; 2 to IPM, **TE**, and **SUT**; 1 to **RIF** and CIP (7) ^C^	[124]
Freshwater and wastewater/Eastern Cape Province, South Africa	*P. fluorescens* (*P. fluorescens*)	Resistance to **P**, **OX**, **CM**, **VAN**, **TMP**, and **RIF**; varied resistance rates to **CF**, **CTX**, **SAM**, and FM ^C^. Detection of *bla*_TEM_ in 57.14% of isolated *P. fluorescens* (14.28% and 31.25% of isolates in freshwater and wastewater, respectively)	[109]
Liver of wedge sole fish *Dicologlossa cuneate*/Coast of Spain	*P. baetica* a390^T^ (*P. koreensis*)	Resistance to **AM** ^C^ Tolerant to **OT** ^B^ Detection of orthologs of MexAB–OprM and MexEF–OprN RND efflux pumps	[125,126]
Coastal waters/Kaštela Bay, Croatia	*P. fluorescens* (*P. fluorescens*)	First report of a chromosomally located *bla*_TEM-116_ in *P. fluorescens*. 70 (of 185) isolates were MDR, with the highest rates for **CTX**, CAZ, MEM, ATM, and **TE** ^E^	[108]
Soils under distinct management (with or without manure/antibiotic history)/Masuria, Warka, and Lesznowola, Poland	*P. jessenii* (*P. jessenii*), *P.* *mandelii*, and *P. fluorescens* (*P. fluorescens*)	Tetracycline (*tet*-like), erythromycin (*erm*-like) and streptomycin (*aac*) resistance genes Detection of integrase, recombinase, and resolvase High MICs for **TE**, STS, and **E** ^B^	[127]
Treated wastewater/Puck Bay, Poland	*P. protegens* (*P. protegens*)	Resistance to TIM, CAZ, FEP, and ATM (1) ^F^	[128]
Small colony variant isolated from biofilm cultures of rhizosphere colonizing *P. chlororaphis* strain 30-84	*P. chlororaphis* strain 30-84	Resistance to **K** and PIP (one small colony variant) ^A^	[129]
Danube River water/Multinational	*P. fluorescens* (*P. fluorescens*)	All eight isolates tested were resistant to CAZ, six to MEM, four to CIP, three to IPM, two to TZP and/or FEP, and one to GM or LVX (eight) ^C^ Modified Hodge test was positive for carbapenemase presence in isolates resistant to MEM and IPM	[60]
*Halimione portulacoides* tissue samples from a metal-contaminated estuary/Ria de Aveiro, northwest coast of Portugal	*P. koreensis* (*P. koreensis*); *P. simiae* (*P. fluorescens*); *P. migulae* (*P. mandelii*), and *P. fragi* (*P. fragi*)	The most common resistance phenotypes included **AM**, **AMX**, **AMC**, and **CTX**. *P. koreensis* (10), *P. simiae* (5), *P. migulae* (1), and *P. fragi* (1) ^C^	[26]
Treated wastewater/Germany	*P. fluorescens* (*P. fluorescens*)	Resistance to β-lactams (**P**, **O CLO**, **CF**, **CZ**, **AMX**, **CRO**, and CB); aminoglycosides (**K**, **NEO**, CAP, AN, GM, SIS, NB, and SPT); **E**, **RIF**, **LIN**, **VAN**, **C**, **BLE**, varied **sulfonamides** and **tetracyclines**; NA, OFL, LOM, and PB (1) ^D^	[130]
Peaty soil from biological pesticide sewage treatment plant/Jaworzno City, Silesia district, Southwestern Poland	Description of *P. silesiensis* strain A3^T^ (*P. mandelii*)	Resistance to ATM, **RIF**, **VAN **^G^ Resistance to **AM** ^B^	[131]
Water from Del Rey Lagoon (DRL). Lower Ballona Creek watershed/Los Angeles County, California, USA	*P. fluorescens* and *P. rhodesiae* (*P. fluorescens*)	Resistance phenotypes included **AM**, **CTX**, **TE**, **E**, **S**, STS, NA, and CIP for *P. fluorescens* (4) and **CTX**, **E**, **TE**, **S**, STS, NA, and CIP for *P. rhodesiae* (1) ^C^	[132]
Leaves of the Ni hyperaccumulator *Alyssum serpyllifolium* (subsp. malacitanum) grown in serpentine soils (high concentrations of heavy metals)/Bragança, Portugal	Drought-resistant *P. azotoformans* strain ASS1 (*P. fluorescens*)	Resistance to **P**, **AM**, **C**, and STS (1) ^C^	[65]
Red fox (*Vulpes vulpes*) feces/Northern Portugal	*P. fluorescens* (*P. fluorescens*)	Resistance to **AMX**, **AMC**, **CF**, **FOX**, **CTX**, TIC, TIM, IPM, ATM, **E**, **TE**, **C**, **SUT**, and FOS ^C^ Resistance to IPM and CIP on biofilm removal. Detection of *bla*_OXA-aer_ (1)	[92]
Fruits and leaves of sick *Citrus sinensis* cv. ‘Valencia Late’ and *Citrus limon* cv. ‘Eureka’/Tunisia	*P. kairouanensis* strains KC12^T^, KC17, KC20, KC22, KC24A, KC25, and KC26; *P. nabeulensis* strains E10B^T^, E10AB, E10CB1, and Iy3BA (*P. fluorescens*)	All strains displayed resistance to **OX**, **CF**, **CZ**, **CXM**, **AMX**, and TM ^C^ MDR phenotype among the strains	[133]
Stream waters and effluents from urban wastewater treatment plants/Central Italy	*P. koreensis* (*P. koreensis*)	Resistance to **AM** and **CTX** ^B^ High MIC values for **AMP**, **CZ**, and **ETP** ^A^ Resistance to CL (5) ^A^ Detection of *bla_AmpC_* in two (of five) isolates	[29]
Roots of *Odontarrhena obovata* on copper-influenced soil/Chelyabinsk region, Russia	Copper tolerant *P. lurida* strain EOO26 (*P. fluorescens*)	Resistance to **AM**, **TE**, **C** and **P **^C^	[84]
Wastewater/Kwara, Nigeria	*P. fluorescens* (*P. fluorescens*)	Resistance to CAZ, **CXM**, **CFM**, OFL, CIP, FM ^C^ The same isolate, when plasmid-cured, was not resistant to CFM, OFL, CIP, and FM	[134]
River, stream, lake, and sewage water samples/São Paulo state and Brasília, Brazil	Heavy-metal resistant *P. saponiphila (P. chlororaphis)*	Resistance to TZP, **CRO**, **CTX**, CAZ, IPM, MEM, FEP, ATM (10, 11, 11, 8, 2, 2, 5 and 7 of 13, respectively). Resistance to **TE** (11); **C** (10); CIP (2); LVX (4) and NOR (5); GM (1) and TM (1). ^A^ First report of *bla_GES_*, *qnrS*, *aac(3′)-IIa,* and *tetB* in *P. saponiphila*.	[73]
Marine polypropylene/Øygarden, Norway	Draft genome sequence of *P. protegens* 11HC2	Resistance to **CTX**, **AM**, **C** and **TMP** ^E^ The strain carries a class C β-lactamase, type-B chloramphenicol O-acetyltransferase (*catB*), three distinct copies of dihydrofolate reductase, and a bifunctional aminoglycoside phosphotransferase	[135]

Antimicrobial agents to which *P. aeruginosa* is considered intrinsically resistant are indicated in bold. Antimicrobials in both bold type and underlined are generally recognized as ineffective against non-fermentative gram-negative bacteria. ^T^: Type strain; ^A^: minimum inhibitory concentration (MIC) by broth microdilution; ^B^: MIC by agar dilution; ^C^: disc-diffusion method; ^D^: phenotype microarray; ^E^: MIC by Etest; ^F^: Phoenix Automated Microbiology System (BD Diagnostic Systems, USA); ^G^: Biolog GENIII Microplates. AM: ampicillin; AMC: amoxicillin–clavulanic acid; AMX: amoxicillin; AN: amikacin; ATM: aztreonam; BAC: bacitracin; BLE: bleomycin; C: chloramphenicol; CAP: capreomycin; CB: carbenicillin; CAZ: ceftazidime; CF: cephalothin; CFS: cefsulodin; CIP: ciprofloxacin; CL: colistin; CLO: cloxacillin; CM: clindamycin; CRO: ceftriaxone; CTX: cefotaxime; CXM: cefuroxime; CFM: cefixime; CZ: cefazolin; E: erythromycin; EN: enrofloxacin; ETP: ertapenem; FEP: cefepime; FM: nitrofurantoin; FOS: fosfomycin; FOX: cefoxitin; GM: gentamicin; IPM: imipenem; K: kanamycin; LIN: lincomycin; LOM: lomefloxacin; LVX: levofloxacin; MEM: meropenem; NA: nalidixic acid; NB: novobiocin; NEO: neomycin; OFL: ofloxacin; OX: oxacillin; P: penicillin; PB: polymyxin B; PIP: piperacillin; RIF: rifampicin; S: sulfamethoxazole; SAM: ampicillin–sulbactam; SIS: sisomycin; SPIR: spiramycin; SPT: spectinomycin; STS: streptomycin; SUT: sulfamethoxazole–trimethoprim (or cotrimoxazole); TE: tetracycline; OT: oxytetracycline; TIC: ticarcillin; TIM: ticarcilli–clavulanic acid; TM: tobramycin; TMP: trimethoprim; TZP: piperacillin–tazobactam; VA: vancomycin;. MDR: multidrug resistant.

**Table 3:** *P. fluorescens* genomes harboring transferable antimicrobial resistance determinants.

				Antimicrobial Resistance Genes						
Accession Number	Taxon ID	Isolation Source	Isolation Country	Aminoglycosides	β-Lactams	Phenicol	Fosfomycin	Sulfamethoxazole	Tetracycline	Other
GCA_000801855.1	*P. fluorescens*	sputum of an individual with cystic fibrosis	USA						*tet(G)*	
GCA_001021695.1	*P. fluorescens*	sputum of an individual with cystic fibrosis	USA	*aac(3)-IIIb*					*tet(B)*	
GCA_001542715.1	*P. fluorescens*	mozzarella cheese	Italy	*aph(3″)-Ib*; *aph(6)-Id*						
GCA_004614275.1	*P. fluorescens*	root	Poland						*tet(A)*	
GCA_900636635.1	*P. fluorescens*	respiratory tract	-	*aph(3′)-Iib*	*bla_OXA_-50*; *bla_PAO_*	catB7	fosA			*crpP*
GCA_004102685.1	*P. azotoformans*	chickpea rhizosphere grown in saline soil	India	*ant(3″)-Ia*				*sul1*		*qacE*
GCA_003851525.1	*P. synxantha*	wheat rhizosphere	USA						*tet(A)*	
GCA_003852025.1	*P. synxantha*	wheat rhizosphere	USA						*tet(A)*	
GCA_008632315.1	*P. veronii*	soil	Svalbard	*aph(3″)-Ib*; *aph(6)-Id*						
GCA_014076455.1	*P. migulae*	biofilm reactor	China	*aadA15*; *aph(3″)-Ib*; *aph(6)-Id*		*cmlA1*; *floR*		*sul1*	*tet(G)*	*qacE*
GCA_002177125.1	*P. koreensis*	lake soil	India	*ant(3″)-Ia*				*sul1*		*qacE*
GCA_003666515.1	*P. protegens*	soil	Netherlands	*aph(3′)-Ia*		*catA1*				
GCA_004212425.1	*P. moorei*	activated sludge	Poland						*tet(A)*	
GCA_000282975.1	*P. psychrophila*	activated sludge sample	China	*ant(2″)-Ia*; *aph(3″)-Ib*; *aph(3′)-XV*; *aph(6)-Id*		*catB3*; *floR*			*tet(G)*	*qacE*
GCA_001043065.1	*P. helleri*	raw milk	Germany	*aph(3″)-Ib*; *aph(6)-Id*					*tet(A)*	
GCA_001050345.1	*P. fildesensis*	Antarctic soil	Antarctica	*aph(3′)-Ia*					*tet(C)*	
GCA_001594225.2	*P. glycinae*	cotton field	USA	*aph(3′)-Ia*						

Among 619 genomes of isolates belonging to the *P. fluorescens* complex available on the NCBI Public Database in November 2021 with source metadata,17 carried acquired resistance mechanisms.

## 4. Final Considerations

The trends recognized in this review support the idea that antimicrobial resistance is a natural phenomenon. Nevertheless, human-impacted sites allow environmental isolates to acquire clinically relevant ARGs. Despite differences in the number of samples and the antimicrobial agents analyzed, the data in Table 1 and Table 2 suggest that MDR phenotypes are abundant in both natural and human-impacted environments. In general, few studies have reported genotype data. However, this situation will likely be improved in the following years as sequencing and whole-genome approaches become more widespread. The characterization of antimicrobial susceptibility phenotypes alongside modern molecular biology techniques will provide key insights into the resistance profiles of *P. fluorescens*.

This review sought to compile the scattered literature on the many different but closely related species of the *P. fluorescens* complex. The data summarized herein contribute to recognizing resistance profiles that are probably associated with intrinsic properties, indicating the features that might be predicted as new or acquired. Considering the “one health” perspective on antimicrobial resistance, the information summarized in this review enables the characterization of the roles of different *Pseudomonas* species as actors in the environmental chain of transfer and maintenance of resistance determinants. Our analysis of the assembled literature also highlighted the importance of developing efficient sewage and wastewater management approaches, as well as the bioremediation of human-impacted sites, as necessary strategies to delay the evolution of antimicrobial resistance in environmental bacteria.

## Data Availability

Data is provided in this article or Supplementary Materials.

## Supplementary Materials

The following supporting information can be downloaded at: https://www.mdpi.com/article/10.3390/antibiotics11080985/s1, Figure S1: Phylogenomic tree of the 98 species belonging to the *P. fluorescens* complex and heatmap representing the digital DDH estimation (dDDH) between genomes. Black squares delimit the species belonging to the same group. The groups already described in the literature are indicated next to the heatmap; Table S1: Digital DDH estimation (dDDH) values between bacterial species belonging to *Pseudomonas fluorescens* complex. These dDDH values were used to plot the heatmap used on Supplementary Figure S1.
