# Supplementary material for: Pseudomonas fluorescens Complex and Its Intrinsic, Adaptive, and Acquired Antimicrobial Resistance Mechanisms in Pristine and Human-Impacted Sites
Source: Antibiotics (Basel). 2022 Jul 22;11(8):985. doi: 10.3390/antibiotics11080985 (PMC9331890; doi:10.3390/antibiotics11080985)
Supplement: Supplementary file 1 [file antibiotics-11-00985-s001.zip › dDDH_Figure S1.pdf]

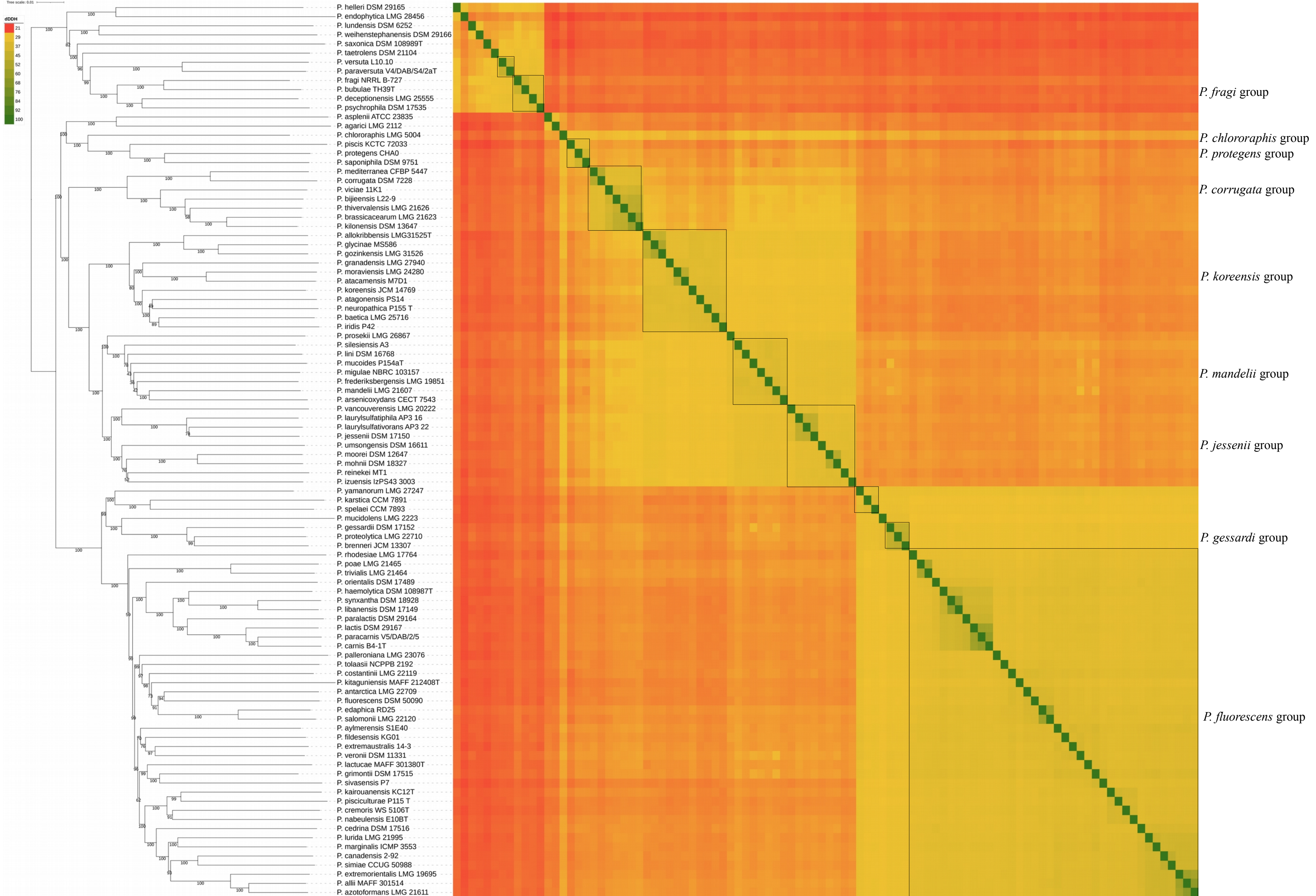
Tree scale: 0.01
dDDH
21
29
37
45
52
60
68
76
84
92
100
P. helleri DSM 29165
P. endophytica LMG 28456
P. lundensis DSM 6252
P. weihenstephanensis DSM 29166
P. saxonica DSM 108989T
P. taetrolens DSM 21104
P. versuta L10.10
P. paraversuta V4/DAB/S4/2aT
P. fragi NRRL B-727
P. bubulae TH39T
P. deceptionensis LMG 25555
P. psychrophila DSM 17535
P. asplenii ATCC 23835
P. agarici LMG 2112
P. chlororaphis LMG 5004
P. piscis KCTC 72033
P. protegens CHA0
P. saponiphila DSM 9751
P. mediterranea CFBP 5447
P. corrugata DSM 7228
P. viciae 11K1
P. bijieensis L22-9
P. thivervalensis LMG 21626
P. brassicacearum LMG 21623
P. kilonensis DSM 13647
P. allokribbensis LMG31525T
P. glycinae MS586
P. gozinkensis LMG 31526
P. granadensis LMG 27940
P. moraviensis LMG 24280
P. atacamensis M7D1
P. koreensis JCM 14769
P. atagonensis PS14
P. neuropathica P155 T
P. baetica LMG 25716
P. iridis P42
P. prosekii LMG 26867
P. silesiensis A3
P. lini DSM 16768
P. mucoides P154aT
P. migulae NBRC 103157
P. frederiksbergensis LMG 19851
P. mandelii LMG 21607
P. arsenicoxydans CECT 7543
P. vancouverensis LMG 20222
P. laurylsulfatiphila AP3 16
P. laurylsulfativorans AP3 22
P. jessenii DSM 17150
P. umsongensis DSM 16611
P. moorei DSM 12647
P. mohnii DSM 18327
P. reinekei MT1
P. izuensis IzPS43 3003
P. yamanorum LMG 27247
P. karstica CCM 7891
P. spelaei CCM 7893
P. mucidolens LMG 2223
P. gessardii DSM 17152
P. proteolytica LMG 22710
P. brenneri JCM 13307
P. rhodesiae LMG 17764
P. poae LMG 21465
P. trivialis LMG 21464
P. orientalis DSM 17489
P. haemolytica DSM 108987T
P. synxantha DSM 18928
P. libanensis DSM 17149
P. paralactis DSM 29164
P. lactis DSM 29167
P. paracarnis V5/DAB/2/5
P. carnis B4-1T
P. palleroniana LMG 23076
P. tolaasii NCPPB 2192
P. costantinii LMG 22119
P. kitaguniensis MAFF 212408T
P. antarctica LMG 22709
P. fluorescens DSM 50090
P. edaphica RD25
P. salomonii LMG 22120
P. aylmerensis S1E40
P. fildesensis KG01
P. extremaustralis 14-3
P. veronii DSM 11331
P. lactucae MAFF 301380T
P. grimontii DSM 17515
P. sivasensis P7
P. kairouanensis KC12T
P. pisciculturae P115 T
P. cremoris WS 5106T
P. nabeulensis E10BT
P. cedrina DSM 17516
P. lurida LMG 21995
P. marginalis ICMP 3553
P. canadensis 2-92
P. simiae CCUG 50988
P. extremorientalis LMG 19695
P. allii MAFF 301514
P. azotoformans LMG 21611
P. fragi group
P. chlororaphis group
P. protegens group
P. corrugata group
P. koreensis group
P. mandelii group
P. jessenii group
P. gessardi group
P. fluorescens group

**Figure S1.** Phylogenomic tree of the 98 species belonging to the *P. fluorescens* complex and heatmap representing the digital DDH estimation (dDDH) between genomes. Black squares delimit the species belonging to the same group. The groups already described in the literature are indicated next to the heatmap.
